# Performance of αSynuclein RT-QuIC in relation to neuropathological staging of Lewy body disease

**DOI:** 10.1186/s40478-022-01388-7

**Published:** 2022-06-22

**Authors:** Sara Hall, Christina D. Orrù, Geidy E. Serrano, Douglas Galasko, Andrew G. Hughson, Bradley R. Groveman, Charles H. Adler, Thomas G. Beach, Byron Caughey, Oskar Hansson

**Affiliations:** 1 Clinical Memory Research Unit, Department of Clinical Sciences Malmö, Lund University, Lund, Sweden; 2 Memory Clinic, Skåne University Hospital, 20502 Malmö, Sweden; 3 LPVD, Rocky Mountain Laboratories, NIAID, NIH, Hamilton, MT USA; 4 Banner Sun Health Research Institute, Sun City, Arizona, USA; 5 Department of Neurosciences, University of California San Diego, La Jolla, CA USA; 6 Veterans Affairs San Diego Healthcare System, San Diego, CA USA; 7 Department of Neurology, Mayo Clinic College of Medicine, Mayo Clinic Arizona, Scottsdale, AZ USA

**Keywords:** Cerebrospinal fluid, Biomarkers, Parkinson disease, Dementia with Lewy bodies, Lewy body dementia, Diagnosis, Autopsy

## Abstract

Currently, there is a need for diagnostic markers in Lewy body disorders (LBD). α-synuclein (αSyn) RT-QuIC has emerged as a promising assay to detect misfolded αSyn in clinically or neuropathologically established patients with various synucleinopathies. In this study, αSyn RT-QuIC was used to analyze lumbar CSF in a clinical cohort from the Swedish BioFINDER study and postmortem ventricular CSF in a neuropathological cohort from the Arizona Study of Aging and Neurodegenerative Disorders/Brain and Body Donation Program (AZSAND/BBDP). The BioFINDER cohort included 64 PD/PDD, 15 MSA, 15 PSP, 47 controls and two controls who later converted to PD/DLB. The neuropathological cohort included 101 cases with different brain disorders, including LBD and controls. In the BioFINDER cohort αSyn RT-QuIC identified LBD (i.e. PD, PDD and converters) vs. controls with a sensitivity of 95% and a specificity of 83%. The two controls that converted to LBD were αSyn RT-QuIC positive. Within the AZSAND/BBDP cohort, αSyn RT-QuIC identified neuropathologically verified "standard LBD" (i.e. PD, PD with AD and DLB; n = 25) vs. no LB pathology (n = 53) with high sensitivity (100%) and specificity (94%). Only 57% were αSyn RT-QuIC positive in the subgroup with "non-standard" LBD (i.e., AD with Lewy Bodies not meeting criteria for DLB or PD, and incidental LBD, n = 23)**.** Furthermore, αSyn RT-QuIC reliably identified cases with LB pathology in the cortex (97% sensitivity) vs. cases with no LBs or LBs present only in the olfactory bulb (93% specificity). However, the sensitivity was low, only 50%, for cases with LB pathology restricted to the brainstem or amygdala, not affecting the allocortex or neocortex. In conclusion, αSyn RT-QuIC of CSF samples is highly sensitive and specific for identifying cases with clinicopathologically-defined Lewy body disorders and shows a lower sensitivity for non-standard LBD or asymptomatic LBD or in cases with modest LB pathology not affecting the cortex*.*

**Supplementary Information:**

The online version contains supplementary material available at 10.1186/s40478-022-01388-7.

## Introduction

The clinical diagnosis of Parkinson’s disease (PD) can be difficult due to its heterogenous presentation and clinical overlap with atypical parkinsonian disorders, especially early in the disease course. Indeed, the diagnostic accuracy for PD has been found to be as low as 73.8% (67.8–79.6) by non-experts but only slightly better by movement disorders specialist with an accuracy of 79.6% (46–95.1) at the initial assessment, particularly when disease duration is less than 5 years [2, 3, 7, 58]. The differential diagnosis of the atypical parkinsonian disorders, multiple system atrophy (MSA), progressive nuclear palsy (PSP), and corticobasal degeneration (CBD) are even more difficult, with generally acceptable specificity but low sensitivity [38, 45].

There are to date no disease-modifying therapies available in PD. New disease-modifying therapies are likely to be most efficient early on in the disease process, before neuronal damage is irreversible [64]. The lack of clear and reliable biomarkers that can identify individuals with PD has been considered a great barrier to the development of disease-modifying treatments [19]. There is thus an urgent need for early and accurate biomarkers for Parkinson’s disease.

Although results consistently have shown decreased levels of CSF unmodified α-synuclein (α-syn) in PD, but also PD with dementia (PDD), dementia with Lewy bodies (DLB), and MSA compared with controls, the reduction is modest, with a broad overlap with controls, and subsequently has failed to adequately discriminate between PD and controls [33, 51, 53], hampering its usefulness in clinical trials and practice.

Ultrasensitive seed amplification assays are methods originally developed for the detection of misfolded prion proteins and prion-like proteins. Over the last several years αSyn RT-QuIC and related α-syn seed amplification assays have emerged as possible methods for detecting misfolded forms of synuclein in CSF, exploiting the prion-like propagation mechanism of pathological α-syn aggregates. Previous studies have shown a high sensitivity of > 92% for clinicopathologically verified Lewy body disorders (LBD), i.e. PD, PDD and DLB, compared to controls/non-synucleinopathies, and a specificity of > 95% [5, 14, 27, 61]. The diagnostic accuracy has predictably been somewhat lower in clinical cohorts with sensitivity > 93% and specificity > 82% [14, 32, 44, 52, 62]. Among αSyn RT-QuIC assays, there are those with particularly short overall assay times of ~ 1–2 days, without compromising diagnostic performance [18, 32, 52, 57, 60–62, 71].

In this study we use a rapid αSyn RT-QuIC assay to test CSF samples from participants with clinically diagnosed PD, PDD, MSA, PSP and controls in the longitudinal Swedish BioFINDER study [34] and participants from a well characterized cohort of neuropathologically-verified cases with different brain disorders including LBD cases and controls from the Arizona Study of Aging and Neurodegenerative Disorders/Brain and Body Donation Program (AZSAND/BBDP) [9]. Previous neuropathological studies have mainly compared "ideal" groups of cases, i.e. controls with no LB disease versus neuropathologically verified and clinically-manifest LBD (“standard LBD”: PD, PDD with Alzheimer’s disease, PDD without AD, and DLB). In this study, we investigated not only individuals with standard LBD and controls, but also those with non-standard LBD (i.e., the cases with Lewy bodies at autopsy but not meeting clinicopathological consensus criteria for DLB or PD), including cases with Alzheimer’s disease with Lewy bodies (ADLB) not meeting criteria for DLB or PD and cases with incidental Lewy body disease (ILBD). Additionally, we investigated CSF αSyn RT-QuIC results in relation to (i) the LB stage, (ii) the LB density and (iii) the LB distribution in ten selected brain regions irrespective of the clinical and neuropathological diagnosis.

## Methods

### Participants in the BioFINDER cohort

The study was performed at the Clinic of Neurology, Skåne University hospital, Sweden as part of the Swedish BioFINDER Study (www.biofinder.se) [34]. The study participants are primarily recruited from the southern region of Sweden. Patients with PD (n = 50) met the NINDS Diagnostic Criteria for PD [29]. Patients with PDD (n = 14) also met criteria for PDD at baseline [25]. Patients with MSA (n = 15) met the consensus statement by Gilman et al. [30]. Patients with PSP (n = 15) met the criteria according to the report of the National Institute of Neurological Disorders and Stroke–Society for Progressive Supranuclear Palsy International Workshop [47]. All controls (n = 47) underwent cognitive testing and neurologic examination by a medical doctor and individuals with objective cognitive or parkinsonian symptoms were not included. Two individuals initially included as controls converted to clinical LBD during follow-up. One was diagnosed with PD after 5.5 years follow-up and one was diagnosed with DLB after 3.5 years follow-up. Exclusion criteria were i) age above 85 years, ii) presence of generalized malignancy, iii) ongoing or earlier advanced abuse of alcohol or illicit drugs, iv) presence of clinically-diagnosed Alzheimer’s dementia, vascular dementia, frontotemporal lobe dementia, v) presence of severe psychiatric disorders, vi) presence of other severe neurological disease, vii) participation in clinical drug trial within the last 30 days.

All participants gave written informed consent before entering the study. The study procedure was approved by the local ethics committee at Lund University Sweden and conducted according to the Helsinki Declaration.

A thorough medical history was taken and the participants underwent extensive testing. Participants were examined by a physician experienced in movement disorders and a registered research nurse using, among other scales, the Unified Parkinson’s Disease Rating Scale (UPDRS) -3, the Hoehn & Yahr scale and the Mini Mental State Examination (MMSE) [26, 28, 37].

CSF samples were obtained by lumbar puncture in the L3/L4 or L4/L5 interspace with patient non-fasting. The samples were collected in polypropylene tubes and gently mixed to avoid gradient effects.

All samples were centrifuged within 30 min at + 4 °C at 2000 g for 10 min to remove cells and debris, and then stored in aliquots at − 80 °C pending biochemical analysis. The procedure followed the Alzheimer’s Association Flow Chart for CSF biomarkers [13].

### Participants in AZSAND/BBDP cohort

The neuropathology cohort consisted of neuropathologically classified participants (n = 101) from the Arizona Study of Aging and Neurodegenerative Disorders (AZSAND), an antemortem-postmortem donor cohort with dates of enrollment from 2007 to 2019. Autopsies were performed by the Banner Sun Health Research Institute Brain and Body Donation program (BBDP) [9, 11].

Neuropathological diagnosis of PD was based on a combination of established neuropathologic criteria [8, 11, 24] and a clinical diagnosis of parkinsonism. DLB diagnosis was defined as a clinical diagnosis of dementia with an intermediate or high likelihood of DLB by the third meeting of the Dementia with Lewy Bodies Consortium [49].

Cases were classified using the Unified Staging System for Lewy Body Disorders (USSLBD) i.e., cases with LBs present were classified into LB stages: I. Olfactory Bulb Only; IIa Brainstem Predominant; IIb Limbic Predominant; III Brainstem and Limbic; IV Neocortical [8]. Lewy Body (LB) density score was assessed in all cases. LB density score is a semi quantitative score of 0–4 in 10 different brain regions (olfactory bulb and tract, medulla at the level of the 9^th^ and 10^th^ cranial nerve nuclei, pons at the level of the locus ceruleus, amygdala, substantia nigra, transentorhinal area, cingulate gyrus at level just posterior to genu of corpus callosum, middle temporal gyrus, middle frontal gyrus, inferior parietal lobule) yielding a maximum score of 40. Neuronal perikaryal cytoplasmic staining, neurites and puncta were considered together, using the templates provided by the Dementia with Lewy Bodies Consortium [49]. The immunohistochemical method used an antibody against phosphorylated synuclein, as previously described[8].

Cases were classified as having PD or DLB, or ILBD (incidental Lewy body disease) in the case of controls without parkinsonism or dementia, when Lewy bodies were present on neuropathological examination but cases did not meet clinicopathological diagnostic criteria for either PD or DLB.

PSP, CBD and MSA were diagnosed according to previously published criteria [22, 23, 31, 36]. Neuropathological diagnosis of AD was based on National Institute on Aging–Reagan Institute (NIA-RI) criteria [1] which are dependent on the Consortium to Establish a Registry for Alzheimer disease (CERAD) neuritic plaque and Braak (neurofibrillary tau-tangle) stage [15]. National Institute on Aging-Alzheimer’s Association criteria[39] were not used due to many cases who had autopsy prior to these newer criteria. The major difference between the two sets of criteria is the addition of Thal amyloid phase to the newer criteria but this may not improve clinicopathological correlations[65]. Controls were cases without dementia or parkinsonism during life and without a major neuropathological diagnosis.

Histopathological scoring was performed blinded to clinical and neuropathological diagnosis. Amyloid plaque and neurofibrillary tangle density were both graded and staged at standard sites in frontal, temporal and parietal lobes and hippocampal CA1 region and entorhinal region using a semi-quantitative score of 0–3 based on the CERAD templates, yielding a total score of 15 [9, 50]. Immunohistochemical staining for phosphorylated TDP-43 was performed in 38 of the 101 cases as previously described [4, 9].

Post mortem CSF was drawn from the lateral ventricles through the corpus callosum prior to removing the brain with precautions taken to minimize contamination. The CSF was then ejected into 15 mL disposable polyethylene tubes. CSF was centrifuged at 2,000 g for 10 min at 24 C and supernatants were aliquoted into 0.5 mL polyethylene microcentrifuge tubes and stored frozen at − 80 °C [9]. Mean post mortem interval was 3.9 (ranging from 2.9–4.9) hours.

Whereas the BioFINDER CSFs were collected from living patients by lumbar puncture, the AZSAND/BBDP CSFs were collected on autopsy from the brain ventricles. Overall comparisons of BioFINDER and AZSAND/BBDP CSFs from PD cases where all replicate reactions were positive indicated that the post-mortem CSFs had slightly higher (22%) mean total protein concentrations, (measured by Pierce Rapid Gold BCA Protein Assay Kit following manufacturer recommendations; ThermoFisher), slightly lower (17%) mean RT-QuIC fluorescence maxima, and slightly longer (15%) mean times to threshold (Additional File 1: Supplemental Fig. 1). However, although these differences in the means are statistically significant, they are small, with large overlaps of the two groups for each of these parameters.

All participants had signed written informed consent. Ethical approval was given by Banner Health-designated Institutional Review Boards; currently the Western Institutional Review Board of Puyallup, Washington.

### αSyn RT-QuIC analyses

αSyn RT-QuIC analyses were performed blinded to clinical and neuropathological status and diagnosis of the patient. The preparation of the K23Q recombinant α‐synuclein was done as previously described [63]. The αSyn RT-QuIC analyses was done as previously described [52]. Briefly, reactions were performed in black 96‐well plates with a clear bottom (Nalgene Nunc International). Each well was preloaded with six glass beads (0.8 mm in diameter, OPS Diagnostics). Quadruplicate reactions were seeded with 15 μL of CSF. Prior to the addition of CSF, each RT‐QuIC reaction mix was 85 μL of solution [32] with final reaction concentrations of 40 mM sodium phosphate buffer, 170 mM NaCl, 0.1 mg/mL K23Q recombinant αSyn (filtered through a 100 kD MWCO filter immediately prior to use), 10 μM thioflavin T (ThT) and 0.0015% SDS. The plates were closed with a plate sealer film (Nalgene Nunc International) and incubated at 42 °C in a BMG FLUOstar Omega plate reader for at least 48 h and subjected to cycles of 1 min shaking (400 rpm double orbital) and 1 min rest for at least 48 h. ThT fluorescence measurements (450  ± 10 nm excitation and 480   ± 10 nm emission; bottom read) were taken every 45 min with fluorimeter gain settings adjusted to maintain fluorescence responses within an unsaturated range (in most cases). The fluorescence threshold was calculated individually for each 96‐well plate to account for differences between plate readers. Positive reactions were those exceeding 10% of the maximum value obtained on the same plate from any individual positive reaction. All samples were subjected to a first round of blinded RT-QuIC testing in quadruplicate: samples with 0 positive reactions within 48 h were deemed negative; samples giving 3 or 4 positive wells were considered positive. If samples initially gave 1 or 2 positive wells, most were retested, and, if the number of cumulative positive wells out of the now 8 total replicates was > 25% we called that sample positive; if not, it was deemed negative. However, two CSF samples giving 1 of 4 positive wells in the initial test were not retested due to technical reasons and were deemed negative for the purposes of this study.

### Statistical analyses

Mann–Whitney U test was used for comparison of continuous and ordinal variables between groups and chi-square test for dichotomous variables. p < 0.05 (two-sided) was considered statistically significant. Sensitivity and specificity of the αSyn RT-QuIC results were calculated for diagnoses in the clinical BioFINDER cohort and between the presence/absence of any LB pathology, LB stage, LB density score, LB distribution and clinicopathological diagnosis in the AZSAND/BBDP cohort. Univariate associations between two continuous or ordinal variables were analyzed using Spearman ρ. SPSS (version 27; SPSS Inc., Chicago, Illinois) was used for statistical analyses and figures.

## Results

### Clinical BioFINDER cohort

There were no sex differences between the diagnostic groups and no significant difference between men and women in αSyn RT-QuIC results (Table 1). There was no significant difference in age between αSyn RT-QuIC positive and negative individuals.

**Table 1:** Patient characteristics, BioFINDER cohort

	Control	PD	PDD	MSA	PSP	Controls that converted to LBD
Number	47	50	14	15	15	2
αSyn RT-QuIC positive (%)	8 (17%)	47 (94%)	14 (100%)	5 (33%)	5 (33%)	2 (100%)
Sex Female/Male (% Female)	27/20 (57%)	25/25 (50%)	6/8 (43%)	6/9 (40%)	8/7 (53%)	1/1 (50%)
Age in years	66.6 (8.9)	68.0 (6.9)	71.1 (6.2)	66.6 (6.6)	72.3 (5.5) ^c, d, i^	65.0 (4.2)
MMSE	28.7 (1.3)	28.9 (1.3)	21.9 (3.8)^c, d^	27.3 (2.9)^e, f^	24.8 (4.8)^c, d^	28.5 (2.1)^h^
Disease Duration in years	NA	6.8 (3.8)	16.8 (6.9)^d^	6.9 (2.7)^f^	6.8 (3.8)^f^	NA
UPDRS III	1.9 (2.7)	13.1 (7.9)^a^	38.4 (11.0)^a, d^	44.5 (17.5)^a, d^	47.5 (14.3)^a, d^	0.5 (0.7)^e, h, i, j^
Hoehn & Yahr	NA	2.1 (0.6)	3.3 (0.7)^d^	4.1 (0.89) ^d, h^	4.3 (0.7) ^d, g^	0 (0)^e, h, I, j^

Data is given as mean and standard deviation (SD) except for in dichotomized values. Significances were analyzed using Mann–Whitney or in the case of dichotomized values Chi-square. a) p < 0.001 vs. control; b) p < 0.01 vs. control; c) p < 0.05 vs. control; d) p < 0.001 vs. PD; e) p < 0.05 vs. PD; f) p < 0.001 vs. PDD; g) p < 0.01 vs. PDD; h) p < 0.05 vs. PDD) i: p < 0.05 vs. MSA; j) p > 0.05 vs. PSP

#### CSF αSyn RT-QuIC vs clinical diagnosis

In the clinically diagnosed BioFINDER study, 94% of PD patients and 100% of PDD patients were αSyn RT-QuIC positive compared with 17% (8/47) of controls (not including those who later converted to LBD) and 33% (5/15) of PSP patients. Further, 33% of those with clinically diagnosed MSA (5/15) were αSyn RT-QuIC positive. Interestingly, both individuals who were included as controls but subsequently developed clinically diagnosed LBD (PD and DLB respectively) during follow up had positive αSyn RT-QuIC results.

In this clinical cohort, αSyn RT-QuIC could discriminate between controls and non-demented PD with a sensitivity of 94% (47/50) and specificity of 83%. When including PD, PDD and individuals who later converted to PD, the sensitivity was slightly higher (95%). However, the three αSyn RT-QuIC negative cases had disease duration of ≤ 5 years yielding a sensitivity of 90% for cases with early PD (≤ 5 years), including those who later developed LBD, and 100% in cases with advanced PD, including PDD (> 5) years. Furthermore, αSyn RT-QuIC discriminated between LBD vs. MSA and PSP with a sensitivity of 95% and a specificity of 67%.

### AZSAND/BBDP cohort

There was no significant difference between men and women regarding CSF αSyn RT-QuIC results in this neuropathology-based cohort (Table 2). As expected, there was a strong correlation between LB stage and LB density total score (Rs = 0.990, p < 0.001).

**Table 2:** Patient characteristics, AZSAND/BBDP cohort

	αSyn RT-QuIC positive	αSyn RT-QuIC negative	*p*-value
Number	41	60	
Age at death, Mean (SD)	83 (7.0)	86 (8.6)	**0.002**
Sex Male/Female	31/10	35/25	0.073
Lewy Body positive	38 (93%)	10 (17%)	** < 0.001**
Lewy Body density total score (max 40) Mean (SD)	21 (11) 1 missing	1.05 (2.9)	** < 0.001**
Lewy Body stage			
0	3	50	
I (olfactory bulb only)	1	2	
IIa (brainstem predominant)	2	3	
IIb (limbic predominant)	7	4	
III (brainstem/limbic)	16	1	
IV (neocortical)	12	0	
Total senile plaque density score (0–15) Mean (SD)	8.05 (6.19)	7.16 (6.25)	p = 0.427
Total neurofibrillary tangle density score (0–15), Mean (SD)	7.70 (3.73)	6.95 (2.94)	p = 0.703
TDP-43 pathology Yes/No (missing)	3/10 (28)	7/18 (35)	p = 0.744

Significances are calculated using Mann–Whitney (continuous and ordinal variables) or chi^2^ (dichotomized variables). *P* < 0.05 was considered statistically significant and is given in bold

#### CSF αSyn RT-QuIC status by LB stage, AZSAND/BBDP cohort

**Fig. 1:**
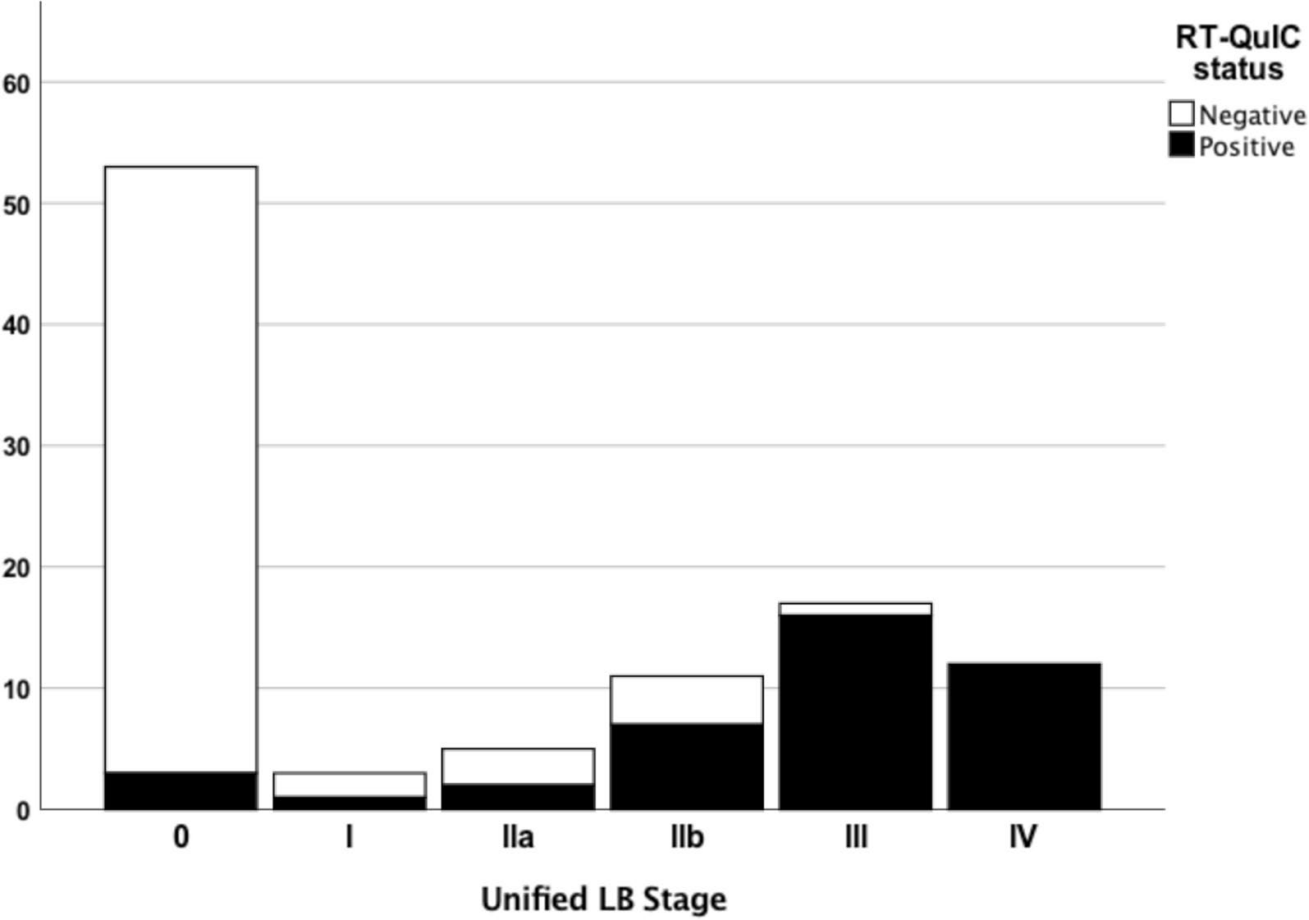
Stacked bar chart depicting αSyn RT-QuIC status by LB stage in the neuropathology-based AZSAND/BBDP cohort. 97% of the cases with LB stage III-IV were αSyn RT-QuIC positive and 93% of the cases in LB stage 0–1 were αSyn RT-QuIC negative. However, only 56% of cases with LB pathology in stage IIa-IIb were αSyn RT-QuIC positive

The frequency of αSyn RT-QuIC positive cases in each LB stage are given in Fig. 1. Of the αSyn RT-QuIC positive cases, 93% (38 out of 41) were LB positive (LB stage I-IV) compared to 17% (10 out of 60) cases in the αSyn RT-QuIC negative group (Table 2). Virtually all of those in LB stage III-IV were αSyn RT-QuIC positive (28 out of 29) and in LB stage 0–1 most were negative (52 out of 56); yielding a sensitivity of 97% and specificity of 93% when comparing LB stage III-IV vs LB stage 0–1 (Table 2; Fig. 1). 2/3 with olfactory bulb only (LB stage I) were αSyn RT-QuIC negative and including LB stage 0 only, the specificity was 94%. However, the sensitivity for detecting LB pathology in stage IIa-IIb was only 56% (9 out of 16). None of the cases with stage IIa-IIb had a clinicopathological diagnosis of PD or DLB. None of the 9 αSyn RT-QuIC positive cases had a parkinsonian clinical diagnosis and although the 16 cases with LB stage IIa-IIb had a mean UPDRS motor score of 16.4 (SD 21.9) there was no significant difference between αSyn RT-QuIC positive and negative cases. Of note is that the single LB stage III case that was αSyn RT-QuIC negative had a relatively low LB density score of 9 compared to a mean of 23.5 (SD 5.1) in all cases with LB stage III.

#### CSF αSyn RT-QuIC status by LB distribution, AZSAND/BBDP cohort

**Fig. 2:**
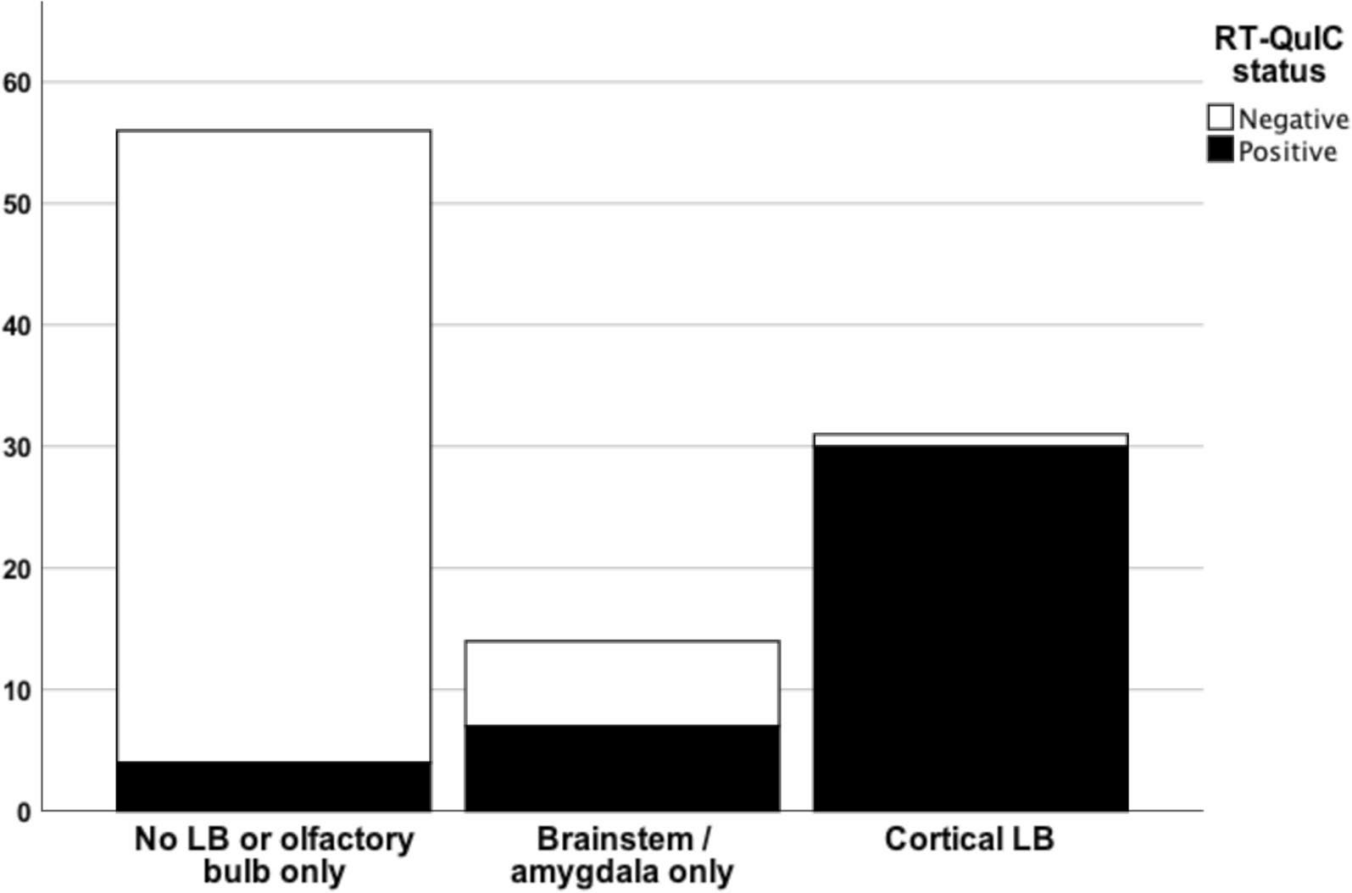
Stacked bar chart depicting αSyn RT-QuIC status by LB distribution in the neuropathology-based AZSAND/BBDP cohort. 97% of cases with LB pathology in the cortex (allocortex and/or neocortex) were αSyn RT-QuIC positive whereas 93% of cases with no LBs or LB restricted to the olfactory bulb only were αSyn RT-QuIC negative. Of the cases with LB pathology in the brainstem and/or amygdala, but with no cortical involvement, 50% were αSyn RT-QuIC positive

When comparing the CSF αSyn RT-QuIC results more directly to the distribution of LB pathology in the brain we found that 30/31 (97%) of cases with LB pathology in the cortex (allocortex and/or neocortex) were αSyn RT-QuIC positive (Fig. 2). The one αSyn RT-QuIC-negative case with cortical LBs was at the LB stage IIb and had a LB density score of only 1 in the cingulum (due to one single LB) and no other cortical involvement. Of the 14 cases with LB pathology in the brainstem and/or amygdala, but with no cortical involvement, 50% were αSyn RT-QuIC positive. (Fig. 2). We next subdivided these 14 cases further into brainstem only (n = 2), amygdala only (n = 7) or those with LB pathology restricted to both brain stem and amygdala (n = 5). We found that 2 out of 2 "brainstem only" were negative, 2 out of 7 "amygdala only" were negative, and 3 out of 5 with "LB pathology restricted to both amygdala and brainstem" were negative.

#### CSF αSyn RT-QuIC status by LB density, AZSAND/BBDP cohort

**Fig. 3:**
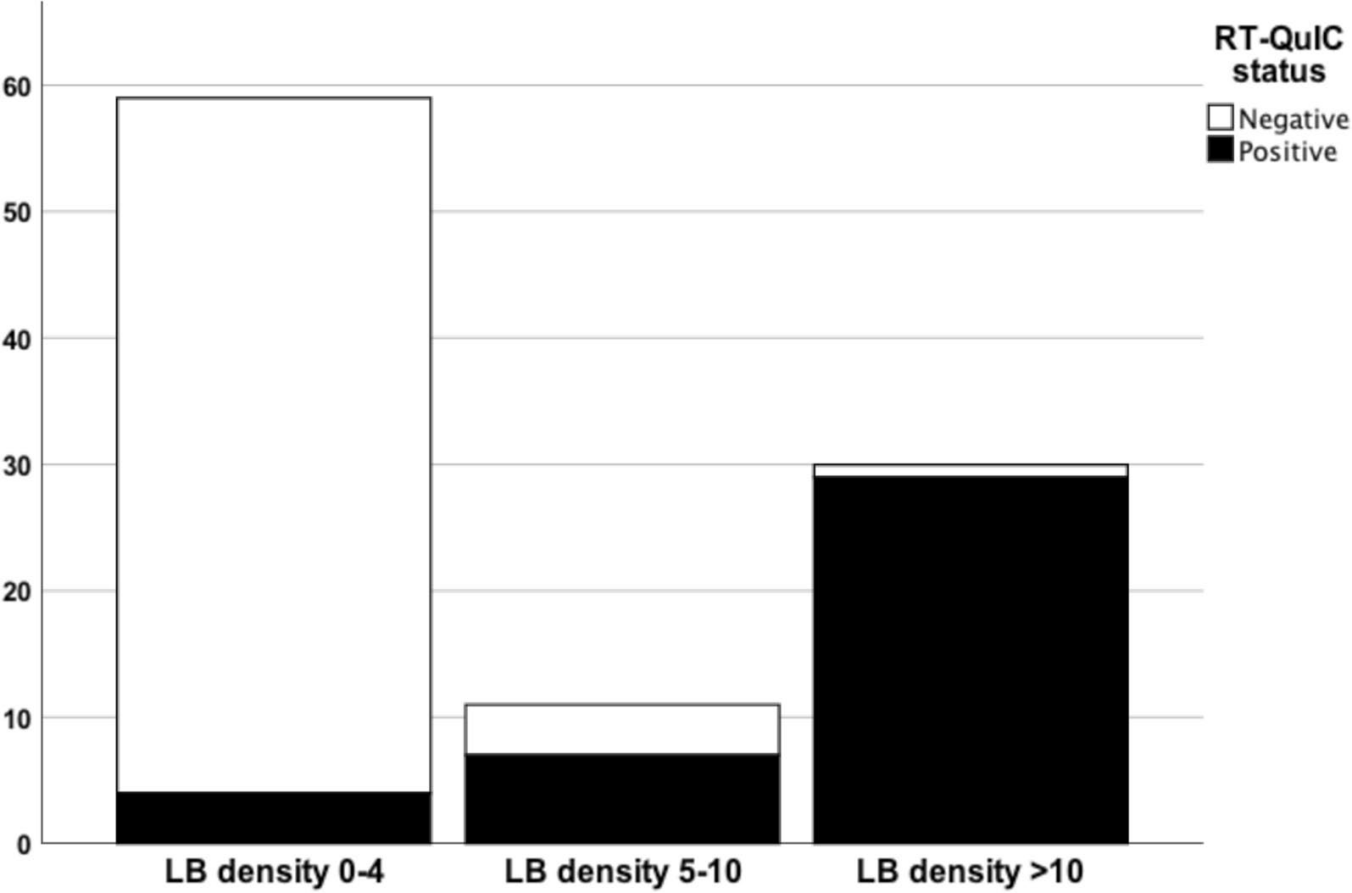
Stacked bar chart depicting αSyn RT-QuIC status by LB density in the neuropathology-based AZSAND/BBDP cohort. 97% of cases with total LB density score > 10 score (established by summing the regional density scores from the ten predefined regions yielding a maximum score of 40) were αSyn RT-QuIC positive. 93% of cases with total LB density score of 0–4 were αSyn RT-QuIC negative. Only 64% of cases with an intermediate LB density score of 5–10 were αSyn RT-QuIC positive

When using the total LB density score (established by summing the regional density scores from the ten predefined regions), the αSyn RT-QuIC negative cases had significantly lower LB density compared with αSyn RT-QuIC positive cases (p < 0.001) (Table 2). αSyn RT-QuIC identified cases with total LB density score > 10 with a sensitivity of 97% (29 out of 30 were positive) and cases with total LB density score 0–4 with a specificity of 93% (55 out of 59 were negative). Of the cases with an intermediate LB density score of 5–10, however, only 64% (7 out of 11) were αSyn RT-QuIC positive (Fig. 3).

#### CSF αSyn RT-QuIC by clinicopathological diagnosis, AZSAND/BBDP cohort

CSF αSyn RT-QuIC identified neuropathologically verified "standard LBD" (i.e. PD, PDAD and DLB with AD; n = 25) vs. no LB pathology (n = 53) with high sensitivity (100%) and specificity (94%). αSyn RT-QuIC thus correctly identified all clinicopathologically confirmed cases with standard LBD. It is worth noting that all 4 cases with DLB also met criteria for AD. In cases with atypical parkinsonian syndromes, i.e. PSP (n = 5), CBS (n = 1) and MSA (n = 1), 6 out 7 were negative. The one αSyn RT-QuIC positive individual with an atypical parkinsonian syndrome had clinicopathological PSP with no evident LBs. It is also worth noting that the one case with neuropathologically confirmed MSA did not have any LB pathology and was αSyn RT-QuIC negative (Table 3).

**Table 3:** Distribution of neuropathological diagnosis in the (AZSAND/BBDP) cohort

Diagnostic category NP diagnosis	N	αSyn RT-QuIC positive	αSyn RT-QuIC negative	Sensitivity	Specificity
**Standard LBD**	**25**	**25**		**100%**	
DLB-AD	4	4		100%	
PD	16	16		100%	
PD-AD	4	4		100%	
PD-PSP*	1	1		100%	
**Non-standard LBD**	**23**	**13**	**10**	**57%**	
ADLB	14	9	5	64%	
ILBD	4	2	2	50%	
LBS-PSP	1		1	0%	
LBS-VaD	2		2	0%	
LBS-VaD-PSP **	1	1		100%	
LBS-Astrocytoma	1	1		100%	
**Lewy Body negative**	**53**	**3**	**50**		**94%**
PSP	2	1	1		50%
PSP-AD	2		2		100%
VaD-CBD	1		1		100%
MSA	1		1		100%
AD	7	2	5		71%
VaD-AD	7		7		100%
Controls	25		25		100%
Other	8		8		100%

^*^Included as PD in statistical analyses

**Microscopic changes of PSP, not included as clinicopathological PSP in statistical analyses. Abbreviations: AD = Alzheimers disease; ADLB = Alzheimer’s disease with Lewy bodies, not meeting criteria for DLB;

CBD = Corticobasal degeneration; DLB = Dementia with Lewy Bodies; LBS = Lewy Bodies, LBD = Lewy Body Disorders; ILBD = Incidental Lewy Body Disease; VaD = Vascular dementia; MSA = Multiple System Atrophy; PSP = Supranuclear Palsy

Bold is for total Standard LBD/Non-standard LBD/Lewy body negative

Of cases with "non-standard LBD" (i.e., AD with Lewy Bodies not meeting criteria for DLB or PD, and ILBD, n = 23), only 57% cases were αSyn RT-QuIC positive (Table 3). αSyn RT-QuIC positive cases with non-standard LBD did not have significantly higher LB stage (p = 0.174) (Table 4) or higher LB density (p = 0.152) compared to αSyn RT-QuIC negative cases. There was no significant difference in LB density in any of the 10 measured brain regions between αSyn RT-QuIC positive and negative cases with "non-standard LBD". Further, there was no difference in UPDRS motor score or MMSE between αSyn RT-QuIC positive and negative cases with "non-standard LBD" (p = 0.755, p = 0.686 respectively).

**Table 4:** Lewy body stage by αSyn RT-QuIC status in Non-standard LBD

	αSyn RT-QuIC positive	αSyn RT-QuIC negative
Stage I	1	2
Stage IIa	2	3
Stage IIb	7	4
Stage III	3	1
Stage IV	0	0

#### CSF αSyn RT-QuIC versus amyloid-β, tau and TDP-43 pathological changes, AZSAND/BBDP cohort

There were no significant differences in total amyloid-β plaque density score or total neurofibrillary tangle density score between αSyn RT-QuIC positive and negative cases. Further, there was no significant difference between the presence/absence of TDP-43 pathology between to αSyn RT-QuIC positive and negative cases (Table 2).

## Discussion

In this study we describe αSyn RT-QuIC testing of CSF from both a clinical cohort and a neuropathological cohort from a longitudinal clinicopathological study. Previous neuropathological studies with αSyn RT-QuIC of CSF have mainly investigated verified clinicopathological diagnoses with LBD vs. cases with no LBD, finding a high diagnostic accuracy in this setting [5, 14, 61], which we can confirm. In the present study we also investigated cases with “non-standard LBD”, i.e. those cases with neuropathological findings of LBD but not meeting criteria for PD or DLB. We found that CSF αSyn RT-QuIC reliably identified cases with LB stage III-IV (Fig. 1), cases with LB disease with cortical involvement (Fig. 2) and cases with high LB brain loads (Fig. 3) and distinguished these from cases with low LB density and no LBs with a high accuracy. However, the sensitivity was less robust when investigating cases with modest LB pathology, defined either as i) low LB stage (IIa-IIb), or ii) limited spread of LBs restricted to the brainstem or amygdala, or iii) intermediate LB brain loads (56%, 50% and 64% respectively), but the number of cases were relatively small.

The diagnostic accuracy was very high for neuropathologically verified cases with standard LB disease vs. cases with no LBs at all with a sensitivity of 100% and a specificity of 94%, which is consistent with previous results [5, 14, 27, 61]. However, in parallel to the results with lower diagnostic accuracy for cases with intermediate LB density or limited spread of LBs, the diagnostic accuracy was also lower in cases with "non-standard LBD", with 57% being αSyn RT-QuIC positive. This is partially in line with the exploratory results by Fairfoul et al. who found up to 31% αSyn RT-QuIC positivity in AD cases with incidental LB, however the number (n = 13) with non-standard LBD in that study was low [27]. In the present study, the αSyn RT-QuIC positive cases with "non-standard LBD" did not have significantly higher LB stage or LB density compared to the αSyn RT-QuIC negative cases with "non-standard LBD". Further, there was no significant difference in LB density in any of the 10 different brain regions in αSyn RT-QuIC positive cases vs. αSyn RT-QuIC negative cases in the "non-standard LBD group". It is therefore not possible to speculate, in this cohort, whether the αSyn RT-QuIC positive cases with "non-standard LBD" would have been at higher risk of developing clinical Lewy body disease. However, there might be a threshold effect, where these cases are close to the threshold of αSyn RT-QuIC detection, yielding a higher variability in the result. This is likely a result of lower total brain (and CSF) load of a-syn seeds in this group of cases with less widespread LB pathology seldom affecting the cortex. Further, the semiquantitative method of obtaining LB regional density score allows for some variability in density scoring. An alternative, but less likely and speculative, explanation is that the α-syn seeds present in the cortex are more prone to induce aggregation of monomeric α-syn.

The presence of more than one pathology is prevalent in neurodegenerative disorders [10, 41, 59] and Aβ and tau pathologies may act synergistically with αSyn pathology influencing the clinical presentation and prognosis in LBD [43]. Further, misfolded αSyn might potentiate aggregation of tau [6, 56]. However, the CSF αSyn RT-QuIC assay could in the present study specifically identify LB pathology without any associations to the load of Aβ, tau or TDP-43 pathology changes, although the number of cases investigated for TDP-43 pathology was low.

In the clinical BioFINDER cohort, we found a high sensitivity (95%) for αSyn RT-QuIC in LBD. However, the specificity in the present study was 83%, which is lower compared to previous results [32, 61]. One can speculate that the lower specificity in the clinical cohort reflects the fact that some of the individuals included as controls could have ILBD, although the present rate would be higher than expected. Supporting the possibility that at least some of these αSyn RT-QuIC positive controls were ILBD is the documented later clinical conversion of two αSyn RT-QuIC positive controls to PD or DLB. The high rate of αSyn RT-QuIC positive controls could also be due to a high sensitivity of the analyses for ILBD; this is, however, at odds with results in the neuropathological AZSAND/BBDP cohort with a low specificity for individuals with ILBD. An alternative explanation could be an increased proclivity for ILBD than expected in the clinical BioFINDER cohort. The controls in the present study were mainly spouses or in some cases 1^st^ degree relatives of individuals with PD (mostly) or atypical parkinsonian disorders. There were, however, no significant differences in the proportions of αSyn RT-QuIC-positive and negative BioFINDER controls that had spouses or 1st degree relatives with LBD. None of the αSyn RT-QuIC positive controls had 1st degree relatives with LBD but one of the αSyn RT-QuIC positive controls that converted to LBD had a sibling with PD.

In our study, 33% of clinical MSA were αSyn RT-QuIC positive and 0/1 of the neuropathologically confirmed cases with verified MSA was αSyn RT-QuIC positive. This is in line with previous studies [61, 70]. It has been proposed that this is due to a different strain of α-syn in MSA [54, 55, 69], yielding a different kinetic profile [66], or no aggregation at all [61], in different αSyn seed amplification assays. Our assay was developed for the detection of seeds of Lewy body disorders rather than MSA, and others have shown better detection of MSA-associated seeds using alternative amplification conditions [48, 62]. The low specificity could also be attributed to clinical misdiagnosis or concomitant LB pathology in MSA[46].

Perhaps more surprising is our low specificity in PSP cases (67%) in the clinical BioFINDER cohort. In our hands, one of the positive PSP samples from the clinical cohort gave 4/4 and one 3/4 positive replicate wells and the others gave 3/8 positive replicate wells. In the 47 positive PD cases, 41 gave 4/4 positive replicate wells, four gave 3/4, one gave 3/8 and one 6/8. All 14 PDD gave 4/4 positive replicate wells. We did not observe any significant difference in the fluorescence intensity in PSP compared to the PD CSF samples. Clinical diagnoses are difficult and inherently uncertain, especially for atypical parkinsonian syndromes and it is possible that the low specificity in the present study may be due to clinical misdiagnoses. However, there were was no significant differences in disease duration, UPDRS motor score, Hoehn & Yahr score, age or sex (data not shown) between the αSyn RT-QuIC positive and negative PSP patients. The presence of multiple pathologies is relatively common in neurodegenerative disorders [42, 59] and the high positivity rate in the clinically diagnosed PSP participants could possibly in part be due to LBD-copathology. This makes the inclusion of a cohort of neuropathologically confirmed cases in the present study all the more important. In clinicopathological cases with PSP, CBD, or MSA we found all but one case to be αSyn RT-QuIC negative; however, the total case number was small.

Given the results in the present study with a very low diagnostic accuracy in cases with relatively low LB load in the neuropathological AZSAND/BBDP cohort (defined as either “non-standard LBD”, intermediate LB density or limited spread of LB pathology), one could speculate that αSyn RT-QuIC analysis of post mortem ventricular CSF has a lower sensitivity for detecting LBD compared with in vivo lumbar CSF with a high sensitivity for clinical LBD. It has been stated that lumbar CSF has significant differences from ventricular CSF but this has been refuted in several articles [16, 17, 35, 67]. It is worth noting that none of the cases with limited spread of LB pathology (stage IIa-IIb) had a clinicopathological diagnosis of PD or DLB, and all clinicopathological PD and DLB cases had LB stage III-IV with at least some cortical LBs. One previous study investigating the neuropathological findings by PD subtype found that 85% of the total cases and 89% of cases with mild-motor predominant PD reached the neocortical stage [20]. It is thus reasonable to assume that most of the clinical, symptomatic LBD cases in the clinical BioFINDER study have LB pathology spread beyond the brainstem and those results are thus not comparable to the results from post mortem ILBD [20, 21]. However, the three αSyn RT-QuIC negative PD cases in the clinical BioFINDER study all had disease duration of ≤ 5 years and it is possible that these had less spread disease, although this is at odds with the αSyn RT-QuIC positive result in the two asymptomatic individuals who converted to LBD. Therefore, based on the results of the present study, it is not possible to say that ventricular CSF is less sensitive compared to lumbar CSF to detect ILBD or cases with relatively low LB load or disease restricted to the brainstem.

Overall, CSF αSyn RT-QuIC has become a promising biomarker assay for the diagnosis of PD and DLB that may vastly improve the diagnostic work up in these patients, but may not greatly improve the detection of individuals at the very earliest stages of the disease. Still, several studies have shown a high sensitivity for αSyn seed amplification assays in prodromal cases [40, 60, 61, 68]. Isolated rapid-eye-movement sleep behavior disorder (iRBD) is a common non-motor symptom in LBD and also a common prodromal symptom of LBD [12]. In a recent longitudinal study on patients with iRBD, 62% converted to PD or DLB during follow-up, of whom 97% were CSF αSyn RT-QuIC positive [40]. In the present study, αSyn RT-QuIC positivity was seen up to 5.5 years prior to diagnosis of the two individuals who later developed LBD in the clinical cohort. However, further *longitudinal* studies are needed to clarify how early in the pre-symptomatic and prodromal phases individuals with early LBD convert to αSyn RT-QuIC positivity in CSF.

The possibility of accurate pre-symptomatic or prodromal diagnosis is especially relevant in coming trials on disease modifying therapies, as these are most likely to be effective if initiated early on in the disease course. αSyn RT-QuIC may be a useful test for at risk iRBD individuals in clinical drug trials to monitor the effect of disease modifying therapies initiated at an early stage.

A limitation to the present study is the lack of neuropathological data in the clinical cohort. However, we also studied αSyn RT-QuIC in a neuropathological cohort which strengthens the results. Further, participants in the clinical cohort are followed over time to ascertain as certain a diagnosis as possible. Indeed, the three αSyn RT-QuIC negative individuals clinical PD diagnosis were followed for 2–5 years in the study, showing typical features for PD including Levo-dopa response but were nonetheless early PD with a disease duration of ≤ 5 years.

In conclusion, the present study confirms that αSyn RT-QuIC has excellent sensitivity and specificity for cases with clinicopathologically verified LB disorders vs those with no LB pathological changes. However, the diagnostic accuracy was poor for cases with LB pathology restricted to non-cortical areas and having an overall low-to-intermediate LB brain load. Further studies are needed to investigate what factors influence αSyn RT-QuIC results in these cases with more limited LB pathological changes and determine at what timepoint in the pre-symptomatic or prodromal stages of LBD that the αSyn RT-QuIC result converts from sub-threshold to clear positivity.

## Supplementary Information

**Additional file 1: Supplemental Fig. 1.** Comparisons of BioFINDER and AZSAND/BBDP CSFs from PD cases where all replicate reactions were positive.

## Data Availability

Anonymized data will be shared by request from a qualified academic investigator for the sole purpose of replicating procedures and results presented in the article and as long as data transfer is in agreement with legislation on the general data protection regulation and decisions by the respective Ethical Review Boards, which should be regulated in a material transfer agreement.
