# Supplementary material for: Performance of αSynuclein RT-QuIC in relation to neuropathological staging of Lewy body disease
Source: Acta Neuropathol Commun. 2022 Jun 22;10:90. doi: 10.1186/s40478-022-01388-7 (PMC9219141; doi:10.1186/s40478-022-01388-7)
Supplement: Supplementary file 1 — Additional file 1: Supplemental Fig. 1. Comparisons of BioFINDER and AZSAND/BBDP CSFs from PD cases where all replicate reactions were positive. [file 40478_2022_1388_MOESM1_ESM.docx]

**Supplementary information**

PERFORMANCE OF αSYNUCLEIN RT-QUIC IN RELATION TO NEUROPATHOLOGICAL STAGING OF LEWY BODY DISEASE

Authors: Sara Hall, MD, PhD^*^; Christina D. Orrù, PhD^*^; Geidy E. Serrano, PhD; Douglas Galasko, MD, PhD; Andrew G. Hughson, MSc; Bradley R. Groveman, PhD; Charles H. Adler, MD, PhD; Thomas G. Beach, MD, PhD; Byron Caughey PhD^**^; Oskar Hansson, MD, PhD**.

* Both contributing equally as first authors

**Both contributing equally as senior authors.

**
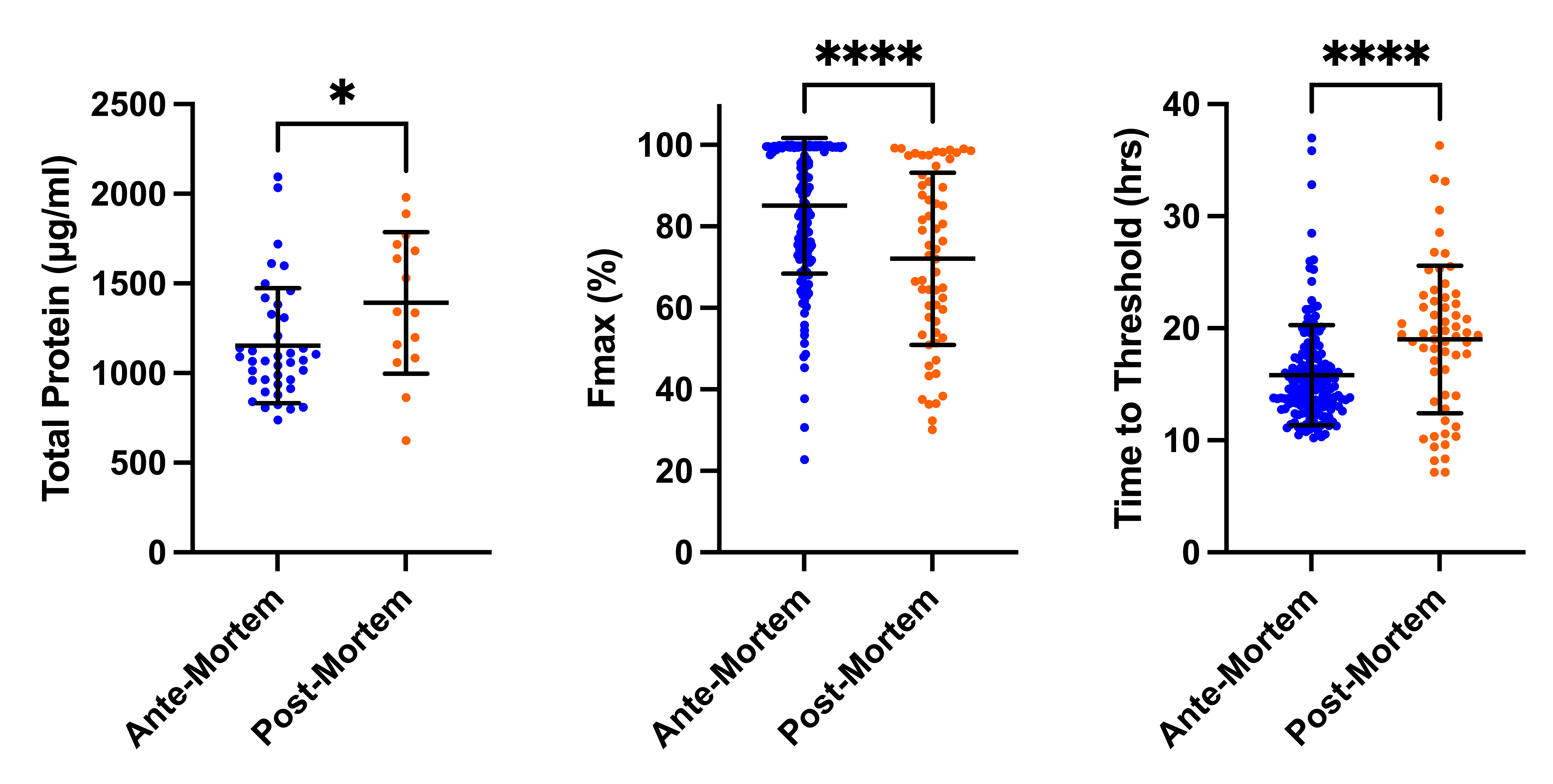
**

**Supplemental Fig. 1.** Total protein concentration and RT-QuIC assay parameters comparison of BIOFINDER (ante-mortem) and AZSAND/BBDP (post-mortem) PD CSFs where all replicate reations were positive. Total protein concentrations were determined using the Pierce™ Rapid Gold BCA Protein Assay Kit (ThermoFisher). Fmax refers to the baseline subtracted maximum thioflavin T fluorescence achieved during an RT-QuIC reaction normalized to the maximum ThT value on each reaction plate. Time to threshold is the reaction time at which fluorescence crossed the positivity threshold (see Methods); total protein data points represent a single assay from individual CSFs. The data points in the other panels indicate individual reaction wells, which were performed in quadruplicate for each CSF sample (i.e., 4 data points per sample).
